# Infecting human brain organoids with FFI or sCJD preserves prion traits regardless of host genotype

**DOI:** 10.1038/s44400-025-00029-9

**Published:** 2025-09-16

**Authors:** B. R. Groveman, S. T. Foliaki, K. Williams, C. D. Orrù, B. Race, G. Zanusso, C. L. Haigh

**Affiliations:** 1 Division of Intramural Research, Laboratory of Neurological Infections and Immunity, National Institute of Allergy and Infectious Diseases, Rocky Mountain Laboratories, National Institutes of Health, Hamilton, MT, USA; 2 Department of Neurosciences, Biomedicine and Movement Sciences, University of Verona, Verona, Italy; 3 These authors contributed equally: B.R. Groveman, S.T. Foliaki

## Abstract

Prion diseases, such as sporadic Creutzfeldt-Jakob Disease (sCJD), are neurodegenerative disorders caused by misfolding of the prion protein (PrP). The D178N mutation in the PrP gene causes Fatal Familial Insomnia (FFI). Here we show that both sCJD and FFI prions can infect human cerebral organoids with or without the D178N mutation, and that the resulting infection is dictated by the inoculating prion and not the host organoid genotype.

Prions are misfolded conformers of the cellular prion protein (PrP). Despite prions’ infectious nature, most human prion diseases are sporadic (~85%), with 5–15% of cases resulting from a genetic mutation in the prion gene. Genetic forms of the disease often present similarly to sporadic Creutzfeldt-Jakob Disease (sCJD) with rapidly progressive dementia associated with neuronal loss, spongiform changes, and gliosis, primarily in the cerebral cortex. The D178N prion gene mutation, when present on the same allele as the most common natural polymorphism PrP-129M, however, results in Fatal Familial Insomnia (FFI), which is primarily characterized by insomnia, cognitive decline, and loss of coordination, associated with thalamic neurodegeneration^1,2^.

Disease-causing PrP mutations are thought to destabilize the native conformation of the protein, enabling it to misfold due to some yet unknown event during aging. The misfolded protein then adopts a more stable, disease-associated form (PrPd). It is thought that variations in the misfolded conformation of PrP encode different disease phenotypes^3^. Once a disease-associated prion is present, it propagates throughout the brain through templated misfolding of native PrP.

Inoculating mouse models with FFI prions demonstrates transmission^4–7^ with pathology that is often distinct from infection with sCJD prions^4^. Several transgenic mouse models spontaneously develop FFI signs and pathology^8–11^, but these vary in their ability to reproduce all aspects of FFI, including transmissibility. It is unclear why some mouse models show production of transmissible prions while others do not; several possibilities are discussed in ref. 10. With the limitations of mouse models, there is a need for models that more closely recapitulate the human host environment.

Our research has previously shown that sCJD prions can be transmitted to human cerebral organoids, which are spheres of neural lineage cells representing cerebral tissue. By five months old, these organoids contain populations of neurons and astrocytes but lack non-neuronal cells and vasculature (limitations of this model are discussed in ref. 12). Sporadic CJD prions propagated in the organoids retain the features of the original inocula^13,14^, demonstrating the clinical signs and biochemical features of their matched, human brain derived, sCJD subtype 1 and 2 (Parchi classification system^15^) when inoculated into mice. These subtypes are defined by their neuropathology and the electrophoretic mobility of protease-resistant prions. Although organoids containing the D178N mutation do not spontaneously generate PrPd, they exhibit multiple phenotypic dysfunctions related to the mutation^16^. Herein, we sought to investigate the ability of FFI and sCJD prions to infect organoids with or without the D178N mutation and what influence the mutation might have on the resulting prion infection.

Patients with FFI have one wild-type and one mutant allele. During the disease, the mutant PrP primarily gets corrupted^17^. We therefore generated cerebral organoids harboring the D178N-129M mutation on both alleles (NN organoids) to isolate the role of the mutant PrP. Similar to the heterozygous organoids, these NN organoids do not develop spontaneous disease^16^. Additionally, since the 129-polymorphism linked to the mutant allele dictates the disease phenotype, we also utilized wild-type organoids (WT) with polymorphism 129M/V (178D/D-129M/V). Because the expressed protein does not contain the D178N mutation, we wanted to see if, following inoculation with FFI prions, the resulting infection would retain FFI characteristics (reminiscent of D178N-129M) or revert to a more CJD-like phenotype (reminiscent of D178N-129V)^1,2^.

Both sets of organoids were inoculated with the same FFI and normal brain homogenate (NBH); however, the WT (129M/V) and the NN (129M/M) were exposed to inocula containing polymorphism-matched sCJD homogenates (MV2 and MM1, respectively^15^). Samples were collected at ~100 and 180 dpi for analysis to look for evidence of disease progression over time. By 180 dpi, neuronal dysfunction was observed in both sets of organoids. Specifically, neuronal burst rates were decreased by nearly 40% in both FFI and CJD-inoculated WT organoids (Fig. 1A, B). These changes were significant, yet less pronounced in the NN organoids (~20% decrease; Fig. 1C, D). In uninfected organoids, the NN mutants display ~65% reduction in burst rate compared to the WT organoids^16^, which could account for the limited reduction seen with infection. Regardless, both WT and NN organoids displayed neuronal dysfunction from infection with either sCJD or FFI.

Proteinase-K (PK) resistant material was detectable at increasing levels from 100 and 180 dpi with MV2 (Fig. 2A, WT [uncropped blots are shown in Supplementary Fig. 2]), but was not detectable in the MM1 (Fig. 2A, NN) inoculated organoids. Typically, Type 1 sCJD does not accumulate as much PK-resistant PrP as Type 2^13,14^ (Supplementary Fig. 1A). However, an increase in total PrP was observed in both sets of CJD-inoculated organoids (Fig. 2B, C; PrP), suggestive of prion accumulation^18^. An increase in astroglia (as measured by GFAP) was also observed in CJD-infected organoids (Fig. 2B, C, GFAP), another hallmark sign of infection. PK-resistant PrP, and an increase in total PrP and GFAP were not observed in the FFI inoculated organoids. This is also not surprising in that these changes take place primarily in the thalamus in FFI patients^19^, a region that is not represented in cerebral organoids. Furthermore, PrPd accumulation is much lower in FFI compared to sCJD (Supplementary Fig. 1A^20^). However, in D178N-129V patients, there is significant cortical involvement^1,2,21^ similar to sCJD, and thus the lack of these markers, particularly in the WT organoids, suggests that the FFI phenotype was retained despite the presence of both 129M and 129V, and the lack of the D178N mutation. The PK-resistant PrP and increased GFAP in the MV2 inoculated organoids were not due to residual inoculum, as total PrP was below levels of detection by western blot in *PRNP* knock-out (KO) organoids and GFAP detection was lower than in WT organoids (Fig. 2D, Supplementary Fig. 3).

Despite the lower accumulation of PrPd in the MM1 and FFI infections being expected based on the lower human brain accumulation of these PrPd, it is a limitation of this data that accumulation did not reach a level that was detectable following PK digest, representative of terminal human disease, such that the electrophoretic profile could be compared with the original brain homogenate. However, it has been demonstrated that prions from different origins may have the same electrophoretic mobility type but display very different disease duration and pathology in mice^22^, thus the electrophoretic mobility alone would be insufficient to indicate whether the original prion type was maintained or changed. Using a more sensitive readout of the properties of the underlying prion, such as differential seeding characteristics, can help to address this.

One differentiating factor between CJD and FFI is their ability to seed replication of misfolded PrP. This has been seen by the difficulty in detecting and diagnosing FFI using Real-Time Quaking-Induced Conversion (RT-QuIC). RT-QuIC uses a small amount of patient tissue and a vast excess of recombinant PrP substrate, exploiting the seeding and self-templating capability of PrPd to rapidly misfold the substrate to detectable levels of aggregation^23,24^. RT-QuIC assays commonly used for the diagnosis of sCJD use a hamster substrate containing amino acids 23 or 90–231. However, at the levels present in diagnostic specimens, these substrates either do not amplify FFI prions or do so with a very low sensitivity (<17%) and slow kinetics compared to sCJD^25,26^. On the contrary, D178N-129V prions can be detected similarly to CJD using the hamster substrates^25,26^. Adapting the assay to use different substrates (e.g., human PrP^27^, hamster-sheep chimera^28^, or truncated bank vole (BV)^29^) has increased the detection of FFI prions in diagnostic specimens, albeit still with lower sensitivity and slower kinetics than sCJD. In brain tissue, however, implementing the use of a BV PrP substrate (amino acids 23–230) increased the kinetics for FFI to match that of sCJD (Supplementary Fig. 1C and ref. 30). Such strategies have been developed to use different RT-QuIC substrates to discriminate between similar prion strains^31,32^. Implementing a similar approach allows us to exploit the differential kinetics of FFI and sCJD prions using hamster 90–231 (Ha90) and BV substrates to discriminate between FFI and sCJD infection of the organoids. Compared with kinetics using Ha90, FFI shows an increase in detection and reaction speed, whereas sCJD displays slower reactions (Supplementary Fig. 1C).

As we observed previously, prion seeding activity in WT organoids inoculated with MV2 increased between the 100 and 180 dpi time points (Fig. 3A, C [RT-QuIC reaction curves are shown in Supplementary Fig. 4]) but declined to mostly undetectable levels in genetically matched organoids where the prion gene has been knocked out (Fig. 3G, H), indicating de novo prion deposition in the WT organoids associated with chronic infection. Using Ha90, an increase from 80 to 100% positive wells and a >3-fold decrease in the time required to cross the designated fluorescence threshold (0.06 ± 0.02 to 0.20 ± 0.02 h^−1^, *p* < 0.0001) was observed (Fig. 3A). The increase in detection from 100 to 180 dpi was similar using the BV, with 100% of the wells scoring positive at both time points, but with faster kinetics at 180 dpi (0.06 ± 0.01 to 0.11 ± 0.02 h^−1^, *p* < 0.0001). End-point dilution analysis showed an increase in seeding dose for both CJD and FFI infected organoids from 100 to 180 dpi (Fig. 3C). In the case of MV2 inoculated organoids, the titer dropped below the level of the inocula at 100 dpi and increased beyond it by 180 dpi. FFI inoculated organoids surpassed the seeding dose of the inocula by 100 dpi and continued to increase to 180 dpi (Fig. 3C), further demonstrating de novo prion accumulation. Although both substrates detected seeding activity in 100% of the wells at 180 dpi, BV exhibited slower aggregation kinetics compared with Ha90 (0.11 ± 0.02 vs 0.20 ± 0.02 h^−1^, *p* < 0.01), consistent with human brain homogenate (Supplementary Fig. 1B). NN organoids were also susceptible to infection with sCJD, displaying 100% detection at both time points and increased kinetics from 100 to 180 dpi (0.12 ± 0.03 to 0.15 ± 0.05 h^−1^ (*p* < 0.05) and 0.06 ± 0.02 to 0.10 ± 0.02 h^−1^, (*p* < 0.001) for Ha90 and BV, respectively) (Fig. 3A). Seeding dose was similarly increased over time, with the MM1 displaying seeding below the levels of the inocula at 100 dpi which increased by 180 dpi (Fig. 3F). FFI infected NN organoids had a seeding dose greater than the inocula by 100 dpi that continued to increase up to 180 dpi (Fig. 3F). Aggregation kinetics were again slower with BV (Fig. 3A; *p* < 0.0001) as with sCJD (Supplementary Fig. 1).

FFI inoculation also resulted in persistent infection of the organoids. Ha90 detected 50% of the wells as positive in the WT organoids at 100 dpi and 62.5% at 180 dpi, mostly with slow kinetics (0.04 ± 0.02 and 0.03 ± 0.01 h^−1^, NS; Fig. 3B). In the NN organoids, only 5 or 10% of the wells were detected at 100 dpi or 180 dpi with the Ha90 substrate. Conversely, with BV 95% and 100% of the wells were positive at 100 dpi in the WT and NN organoids and 100% at 180 dpi with faster aggregation kinetics than with Ha90 (Fig. 3B). The 180 dpi organoids displayed nearly 2-fold faster kinetics compared to 100 dpi (WT: 0.04 ± 0.01 to 0.08 ± 0.02 h^−1^, *p* < 0.0001 and NN: 0.07 ± 0.03 to 0.13 ± 0.03 h^−1^, *p* < 0.0001; Fig. 3B). Seeding dose in both the MM1 and FFI inoculated organoids increased over time, with MM1 showing a drop from the initial inocula followed by a rebound and FFI showing a continued increase over the inocula at both 100 and 180 dpi (Fig. 3F). Together, the increased detection and aggregation kinetics with the BV substrate compared to the Ha90 substrate in the FFI inoculated organoids, and the decrease in aggregation kinetics with the BV substrate in the organoids inoculated with sCJD suggests that in both the WT and the NN organoids that the prions generated with infection maintained the seeding characteristics of the initial inoculum.

Altogether, this data supports that not only are organoids able to propagate both sCJD and FFI prions, retaining the seeding substrate preference of the original infecting inoculum, but also that the inoculating prion is the primary factor driving the formation of the misfolded prions, overriding the presence or absence of the FFI causing mutation. Furthermore, we provide a new model for studying FFI in a human brain tissue homolog.

## Methods

### iPSC and cerebral organoid culture

The production and routine maintenance of the human-induced pluripotent stem cells (hu-iPSCs) used in this study have been described in detail previously^13,16,33^. In brief, WT (178D/D-129M/V; ATCC)^13^, NN (178N/N-129M/M)^16^, and *PRNP* KO^33^ iPSCs were routinely cultured on low growth factor Matrigel in mTeSR1 Plus medium with 5% CO_2_ in a humidified incubator and passaged before colonies started to contact each other.

Organoids were generated from the above iPSCs using the cerebral organoid differentiation kit (Stem Cell Technologies), which is based on the original protocol developed by Lancaster and Knoblich^34^. After differentiation, cultures were maintained in conical flasks on an orbital shaker at 70 rpm in complete maintenance medium: 1× glutamax, 1× penicillin/streptomycin solution, 0.5% vol/vol N2, 1% vol/vol B27 with retinoic acid and 0.5× nonessential amino acids, 0.025% vol/vol insulin, and 0.00035% vol/vol 2-Merceptoethanol in 1:1 Neurobasal:DMEM-F12 medium, under standard incubator conditions (5% CO_2_, 37 °C, humidified).

### Prion infections of human cerebral organoids

Prior to inoculation, cerebral organoids were cultured for 5 months following neural induction to allow for the development and maturation of astrocytes and neurons^35^. Organoid infections were performed as described previously^33^. Briefly, all the culture media were replaced with fresh media containing 0.1% w/v brain homogenates from donors with type 1 (PrP-129M/M) or type 2 (PrP129-M/V) sCJD or FFI. Unaffected brain tissue (“normal brain homogenate”, NBH) was used as a control (obtained from the NIH Neurobiobank at the University of Maryland, Baltimore, MD.). After 24 h, the inoculation media were diluted 1:1 with fresh media and incubated for 6 more days. Following inoculation, organoids were transferred to new vessels containing fresh media.

Seeding dose 50 values for the brain tissues used for inoculation (SD_50_’s; see “RT-QuIC analysis” section) were 7.25, 7.5, and 5.7 logSD_50_’s per mg of neat MM1, MV2, and FFI brain tissue, respectively. Diluting to 0.1% w/v for inoculation would expose the organoids to theoretical logSD_50_’s of 4.25, 4.5, and 2.7 logSD_50_’s per μL of inocula (Supplementary Fig. 1B).

### Neuro-electrophysiology

At ~100 and 180 dpi, individual organoids were plated into 24-well multielectrode arrays (Multi-well-MEA; Multi Channel Systems) with 12 electrodes (700 μm spacing; 100 μm of electrode diameter) per well. Cerebral organoids were embedded onto the electrodes with laminin (Corning) as described in ref. 36. The embedded organoids were incubated unmoved for 24 h to allow strong adherence to the electrodes before recording their electrophysiology and remained in situ throughout the experiment. The local field potential (LFP) sampling at 20 KHz was recorded for five minutes using a Multi-well Screen software (version 1.11.6.0; Multi Channel Systems). A Multi-well Analyzer software (version 1.8.5.0; Multi Channel Systems) was used to filter the LFP with a high-pass (300 Hz) and low-pass (3500 Hz) Butterworth filter and extract neuronal population spikes. A threshold detection method, with 3.7 standard deviations above the mean amplitude, was used to detect spikes. The neuronal burst was detected as a minimum of four spikes firing less than 50 ms apart for a duration of at least 50 ms. The minimal interval between bursts was 100 ms.

### Western blot and proteinase-K (PK) digestion

Following electrophysiological recordings, organoids were recovered from the plates and rinsed with phosphate-buffered saline. All liquid was removed, and the organoids were weighed using a laboratory fine analytical balance in sample preparation tubes, and the tube weight was subtracted. Organoids were homogenized to 10% (w/v) by adding 9× organoid weight volumes of phosphate-buffered saline, grinding with in motorized pestle, and then clearing by centrifugation at 2000 × *g* for 2 min. Homogenates were denatured by boiling for 5 min in 1× sample buffer containing ~6% (v/v) β-mercaptoethanol. Twenty microliters of the samples were loaded onto and resolved using Bolt 4–12% Bis-Tris gels (Invitrogen) and transferred to PVDF membrane (Millipore). The marker was SeeBlue Pre-Stained Protein Standard (Life Technologies). The membranes were probed using anti-PrP 3F4 (Millipore; MAB1563) or anti-GFAP (AbCam; Ab7260) antibodies. The protein bands were visualized using HRP-conjugated secondary antibodies and an ECL chemiluminescent substrate and imaged by an iBright imaging system (Invitrogen). Total protein was measured by staining the membrane with Coomassie blue.

For PK-digestion of both brain or organoid tissue, 15 μL of 10% homogenates were digested in the presence of 1% (v/v) sarkosyl with 50 or 10 μg/ml PK (for brain or organoid tissue, respectively) at 37 °C for 1 h with 400 rpm shaking and then boiled for 5 min in 1× sample buffer. Samples were immediately used for Western blot analysis.

### RT-QuIC analysis

Real-time quaking-induced conversion (RT-QuIC) assays were performed similarly to those reported previously^13,16^. Briefly, the RT-QuIC reaction mix contained a final concentration of 10 mM phosphate buffer (pH 7.4), 300 mM NaCl, 0.1 mg/mL bank vole recombinant PrP23–230 M109I (BV)^30^ or truncated hamster recombinant PrP90–231 (Ha90)^37^, 10 μM thioflavin T (ThT), and 1 mM ethylenediaminetetraacetic acid tetrasodium salt (EDTA). Homogenates were diluted to 0.1% wt/vol, a 10^−3^ dilution, in 0.05% SDS/1× PBS/1× N2 solution. A volume of 98 μL of reaction mix was loaded into a black 96-well plate with a clear bottom (Nunc), and reaction mixtures were seeded with 2 μL of the diluted homogenate for a final reaction volume of 100 μL. The final SDS concentration in the reaction, as contributed by the seed dilution, was 0.001%. For reactions using Ha90 substrate, an additional 0.001% SDS was supplemented into the reaction for a final concentration of 0.002%. The sample dilution was split between two reaction plates, one with BV and one with Ha90 substrate. Reactions were run in quadruplicate for each sample. Plates were sealed (Nalgene Nunc International sealer) and incubated in a BMG FLUOstar Omega plate reader at 42 °C for BV or 50 °C for Ha90 for 40 h with cycles of 60 s of shaking (700 rpm, double-orbital) and 60 s of rest throughout the incubation. ThT fluorescence measurements (excitation, 450 ± 10 nm; emission, 480 ± 10 nm [bottom read]) were taken every ~45 min. Reactions were considered positive if the ThT fluorescence was greater than 10% of the maximum ThT fluorescence value on the reaction plate by the 40 h time cutoff. Results are represented as the inverse of the time required for a reaction to cross the fluorescence threshold. End-point dilution analysis was performed and analyzed using the Spearman–Kärber method to provide estimates of the level of seeding activity or “seeding dose” in a sample. Seeding dose is represented as the number of units giving positive reactions in 50% of replicate reactions, i.e., the 50% “seeding doses” or SD50’s as previously described^23^.

### Statistical analysis

Statistical analyses were performed using GraphPad Prism version 10.0.0 for Windows, GraphPad Software, Boston, Massachusetts, USA, www.graphpad.com. Statistical tests used and number of replicates are described in the figure legends.

## Supplementary Material

Supplementary Info

Supplementary information The online version contains supplementary material available at https://doi.org/10.1038/s44400-025-00029-9.

## Figures and Tables

**Fig. 1 |:**
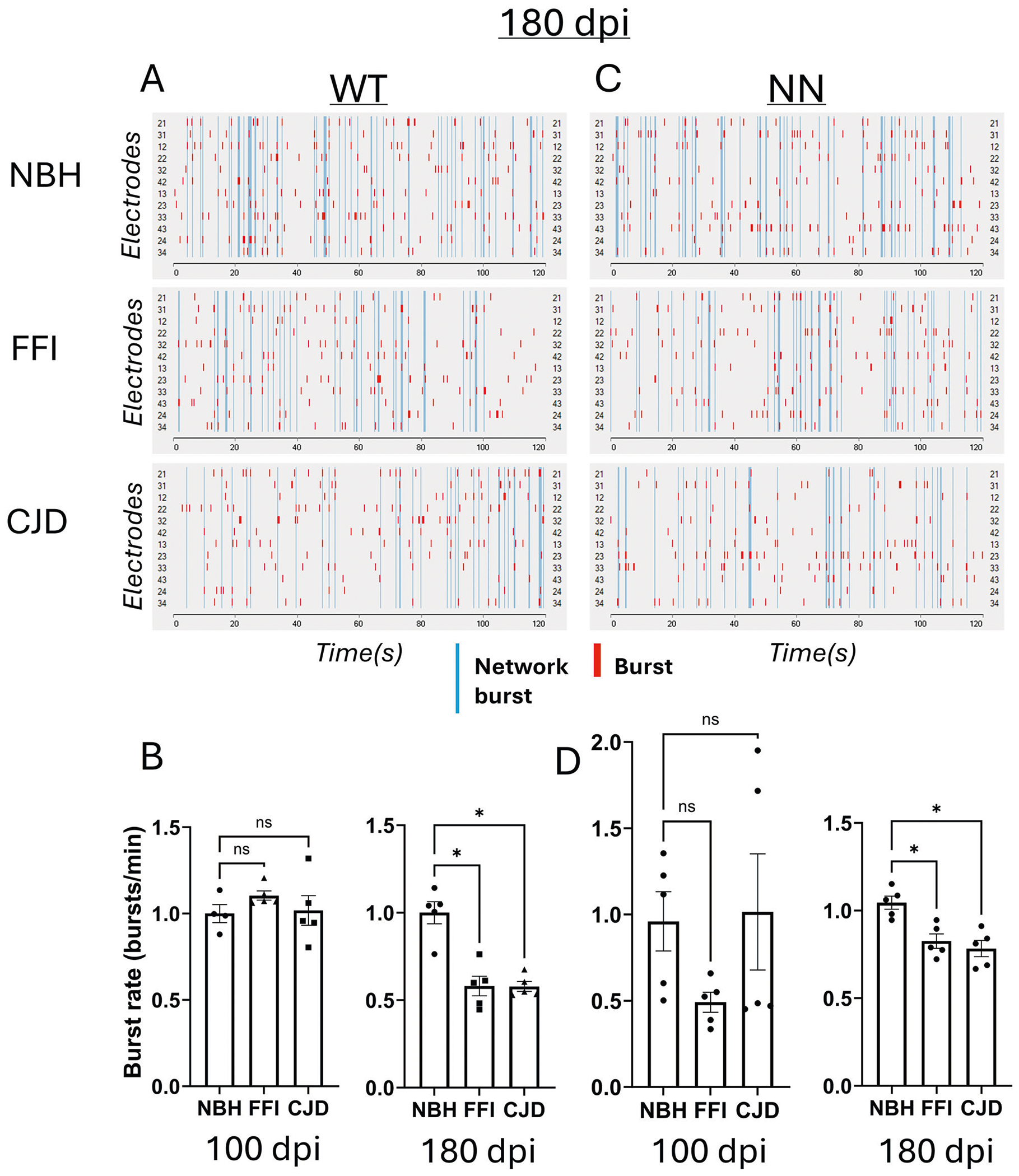
Neuronal dysfunction in infected organoids. Representative raster plots of wild-type (WT) (**A**) or NN (**C**) organoids at 180 dpi after inoculation with normal (NBH), FFI, or CJD brain homogenates. Thick red lines indicate individual bursts, and thin blue lines indicate network bursts. Summary data is shown for WT (**B**) and NN (**D**) organoid recordings for both 100 and 180 dpi. Each marker indicates an individual organoid (*n* = 5 each). Each organoid was run twice, and values were averaged except for WT 100 dpi organoids. *p < 0.05; ns: not significant by Kruskal–Wallis test.

**Fig. 2 |:**
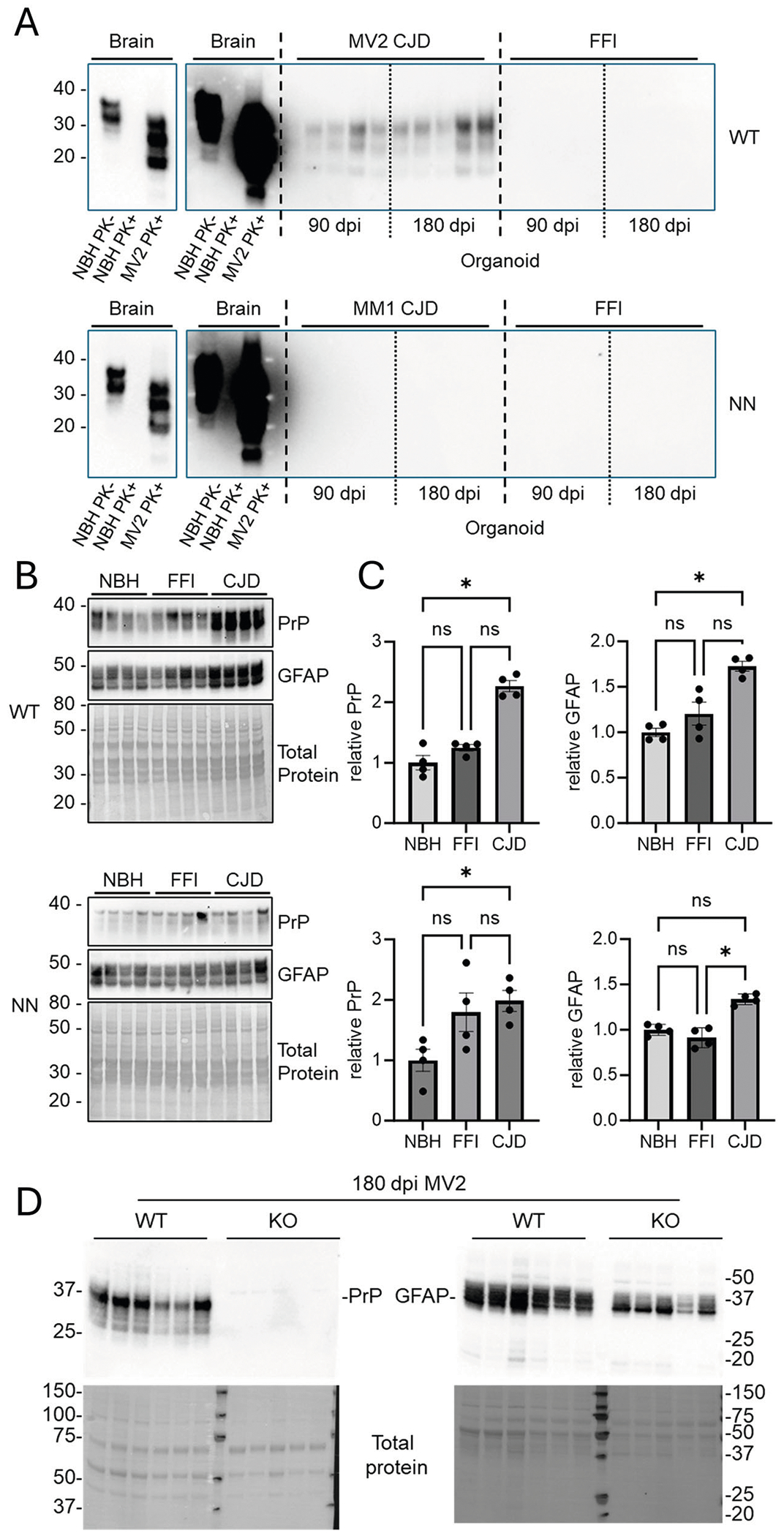
Accumulation of disease-associated markers. **A** PK-digestion of WT (top) or NN (bottom) organoids inoculated with CJD or FFI at 90 and 180 dpi (*n* = 5 for each). Normal (NBH) and CJD brain controls are shown as lower (left) and higher exposures, and are loaded the same on each blot. Comparison of brain tissues used for inoculation can be found in Supplementary Fig. 1. **B** Western blots of total PrP and astrocytes (GFAP) in WT (top) and NN (bottom) organoids at 180 dpi (*n* = 4 each), quantified in (**C**) (*p < 0.05; ns: not significant by Kruskal–Wallis test). Each marker indicated one organoid. **D** Immunoblot of WT and KO organoids inoculated with MV2 sCJD brain homogenate at 180 dpi probed for total PrP (left; 3F4 antibody) or GFAP (right). No residual PrP or gliosis can be observed in the KO organoids as a result of exposure to the inocula. Total protein was observed by Coomassie stain.

**Fig. 3 |:**
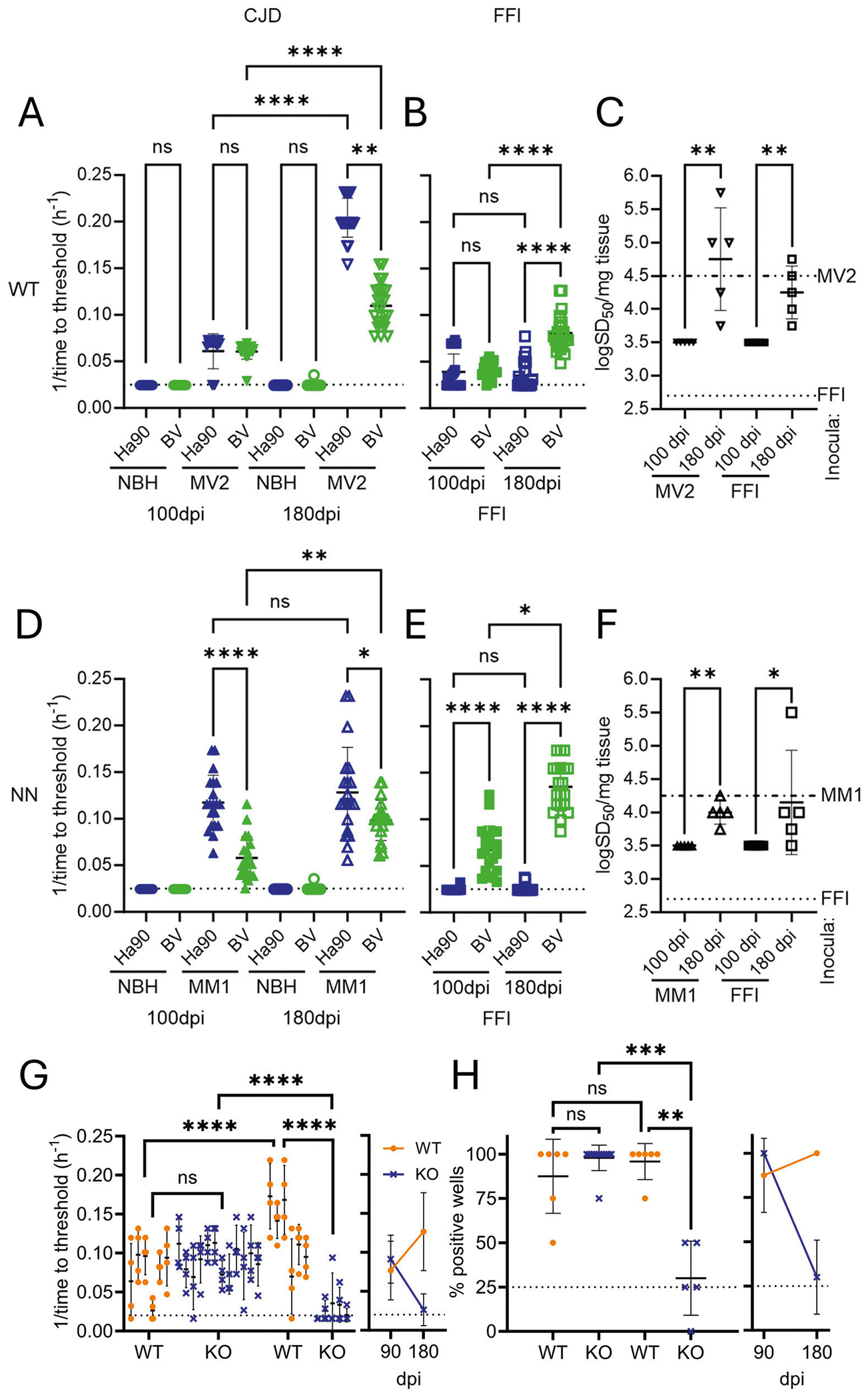
Differential detection of FFI and CJD prions in inoculated organoids by RT-QuIC. Summary data of WT (top panels) or NN (bottom panels) organoids inoculated with NBH, CJD (**A, D**), or FFI (**B, E**) and tested with either Ha90 or BV substrates at 100 or 180 dpi. Each marker indicates individual replicate reaction wells seeded with a 10^−3^ organoid dilution; 4 replicated wells were tested per organoid from 5 organoids per condition (statistical analyses were carried out on the averaged value for each individual organoid). Blue: Ha90 substrate, green: BV substrate; circles: CJD-inoculated, triangles: FFI inoculated; closed markers: 100 dpi, open markers: 180 dpi; *p < 0.05; ***p < 0.001; ****p < 0.0001, ns: not significant by Kruskal–Wallis test. The dotted line indicates the time cutoff of the reaction. **C** and **F** show seeding dose 50 values, displayed as log values, for WT (**C**) or NN (**F**) organoids infected with CJD (triangles) or FFI (squares), at 100 (closed markers) and 180 dpi (open markers), tested with bank vole substrate. The dashed lines indicate the theoretical seeding doses of inocula that the organoids were exposed to (see “Methods” section). The markers show each SD50 for five organoids tested per condition, with 4 replicate reactions averaged per organoid per dilution (statistical analyses were carried out on the averaged value for each individual organoid). *p = 0.0476; **p = 0.0079 by Mann-Whitney test. **G** and **H** RT-QuIC seeding activity in wild-type (WT) and PrP knock-out (KO) organoids inoculated with MV2 sCJD prions. Seeding kinetics (**G**) and total positive wells (**H**) increase from 90 to 180 dpi in WT (orange circles; *n* = 6) but decrease in KO (blue “x”; *n* = 12 for 90 dpi and *n* = 6 for 180 dpi) organoids. The right panels in each show the averaged data, the mean, and the standard deviation. The dotted lines indicate the time cutoff of the reaction (**A**) or the replicate well percent positivity threshold. **A** ****p < 0.0001, ns: not significant by two-way ANOVA. **B** **p < 0.005; ***p < 0.005, ns: not significant by Kruskal–Wallis.

## Data Availability

All data presented herein are present within the manuscript or available from the authors upon reasonable request.
