# Supplementary material for: Infecting human brain organoids with FFI or sCJD preserves prion traits regardless of host genotype
Source: NPJ Dement. Author manuscript; Available in PMC 2025 Oct 4. (PMC12493991; doi:10.1038/s44400-025-00029-9)

**Infecting human brain organoids with FFI or sCJD preserves prion traits regardless of host genotype.**

Groveman, BR^1†^., Foliaki, ST^1†^., Williams, K^1^., Orrù, CD^1^., Race, B^1^., Zanusso, G^2^., Haigh, CL^1^*


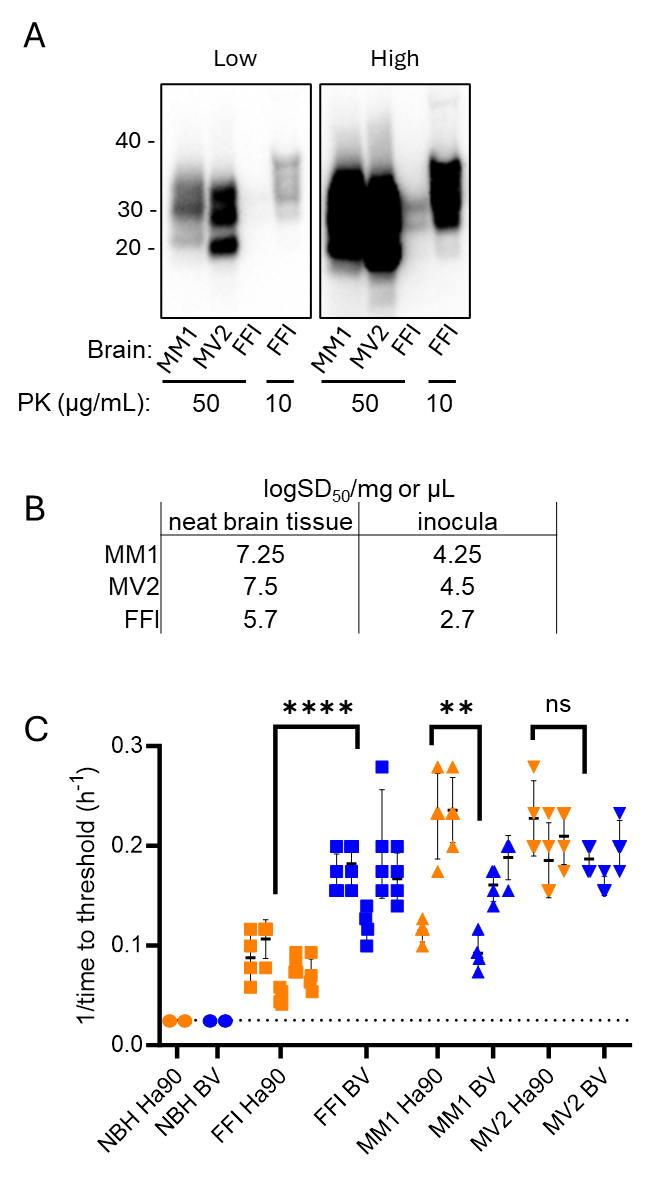


**Supplemental figure 1**: Classification and differential detection of FFI and CJD prions in human brain homogenates. A) detection or protease-resistant PrP in the brain tissues used for inoculation at the typical (50µg/mL) and low (10 µg/mL) proteinase-K (PK) concentrations. Low (left) and high (right) exposures were performed to better visualize the lower levels of PK-resistant PrP in the FFI sample. B) logSD_50_ values for the homogenates, and theoretical logSD_50_ values for the inocula following 1000x dilution in media. C) Human brain tissue dilutions from normal (NBH; circles; n=2), FFI (squares; n=5), MM1 sCJD (upward triangles; n=3), or MV2 sCJD (downward triangles; n=3) donors were tested at 10^-3^ with either Ha90 (orange) or BV (blue) substrates. Each marker indicates the inverse time to threshold of an individual replicate reaction well (n=4 wells per condition), grouped by individual donor brain homogenate. The dotted line indicates the 40h time cutoff used for the reaction. **p<0.005; ****p<0.0001, ns: not significant by two-way ANOVA.

**Supplemental Figure 2:** Uncropped blots.


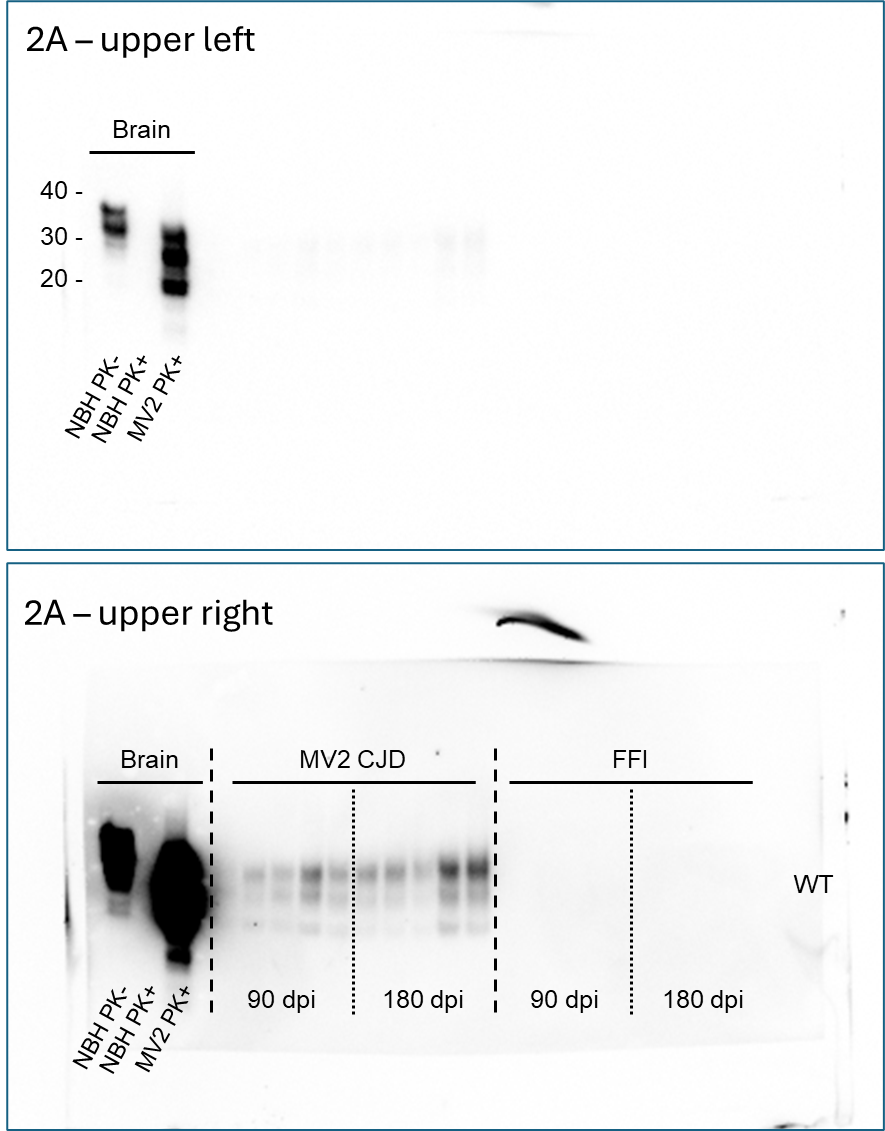


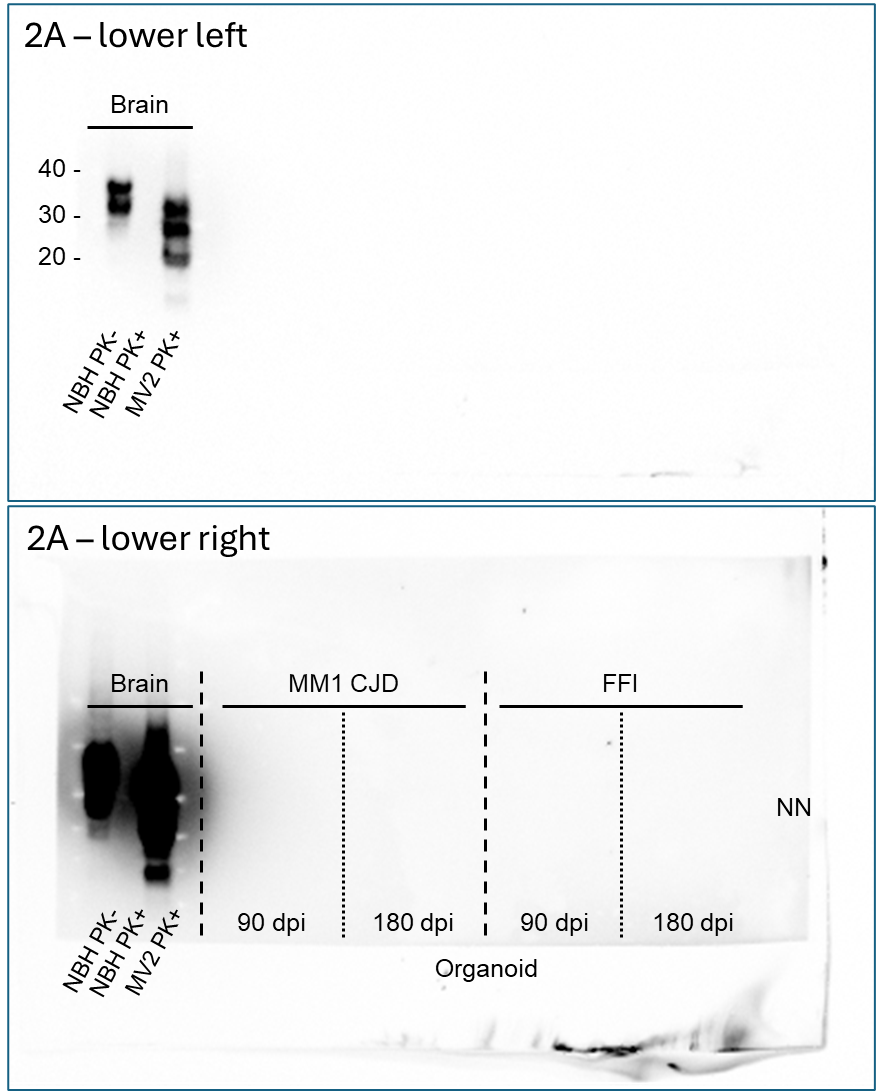


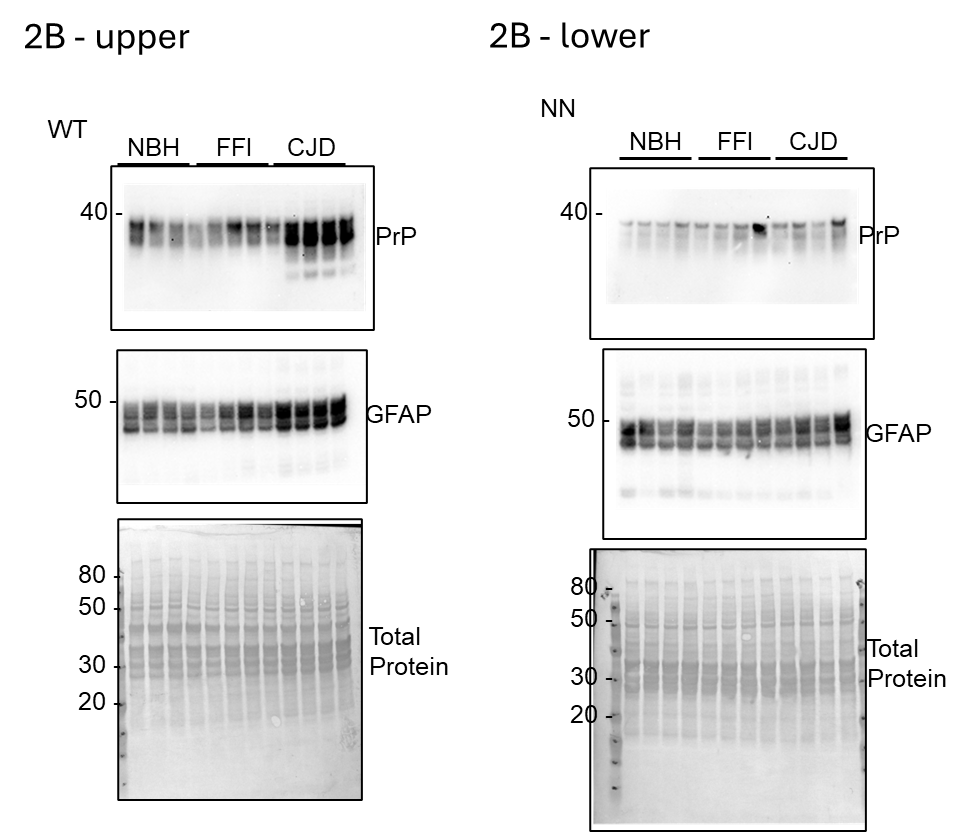


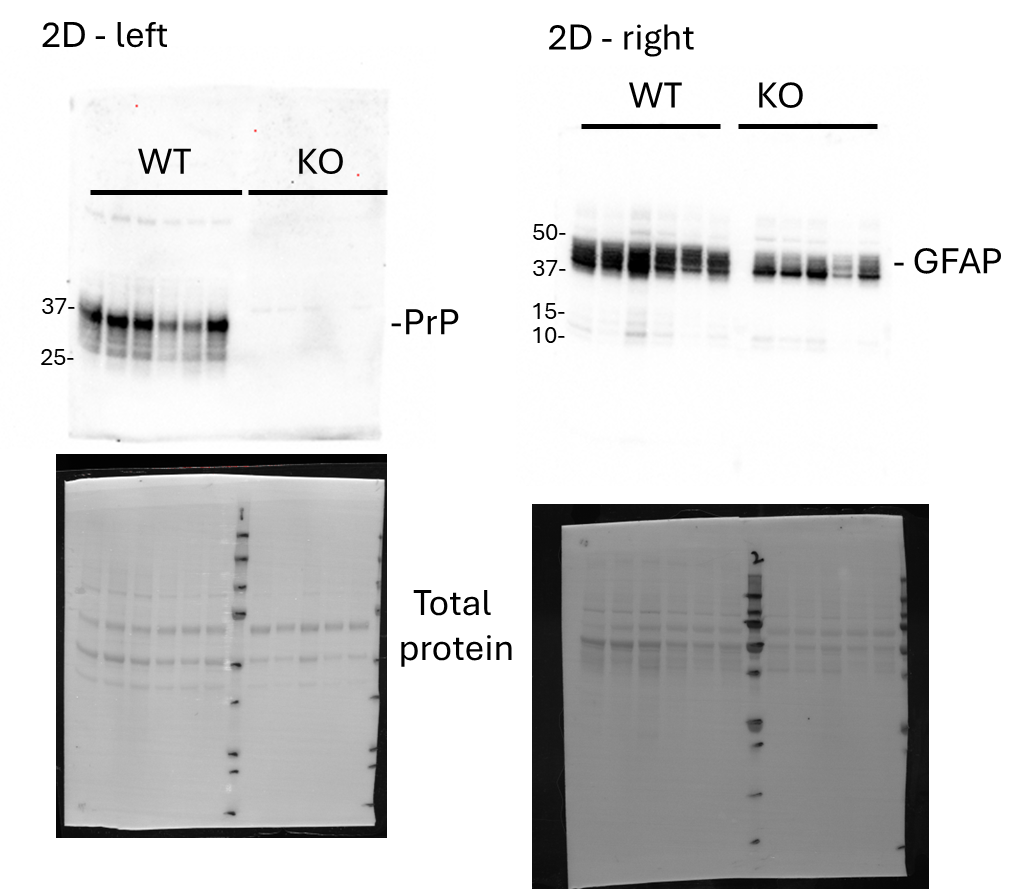


**Supplemental figure 3:** PrP (6H4; brown pigment) immunohistochemistry staining of WT and KO organoids inoculated with normal (NBH) or MV2 sCJD brain homogenate. Blue arrows indicate diffuse residual inocula. Red arrows indicate deposits of de novo PrPd aggregates. Scale bar = 50 µm.

**
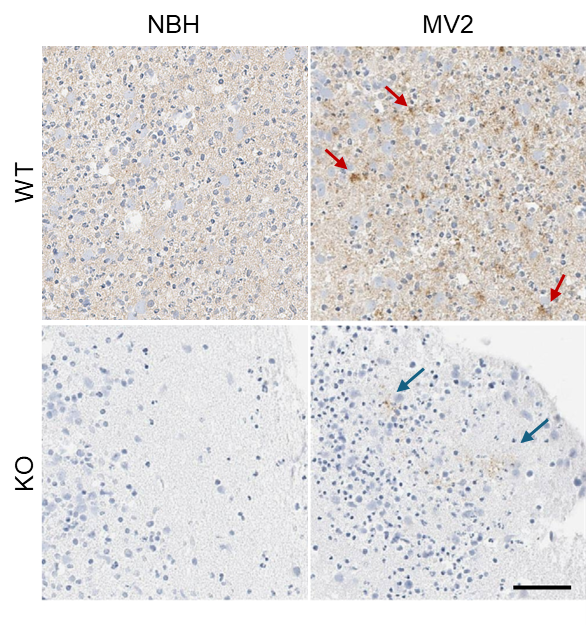
**

**Supplemental Figure 4:** RT-QuIC reaction curves. A) Combined data showing the mean and SEM for the 5 organoids per condition using the bank vole substrate. B) Raw data showing all individual replicate wells.


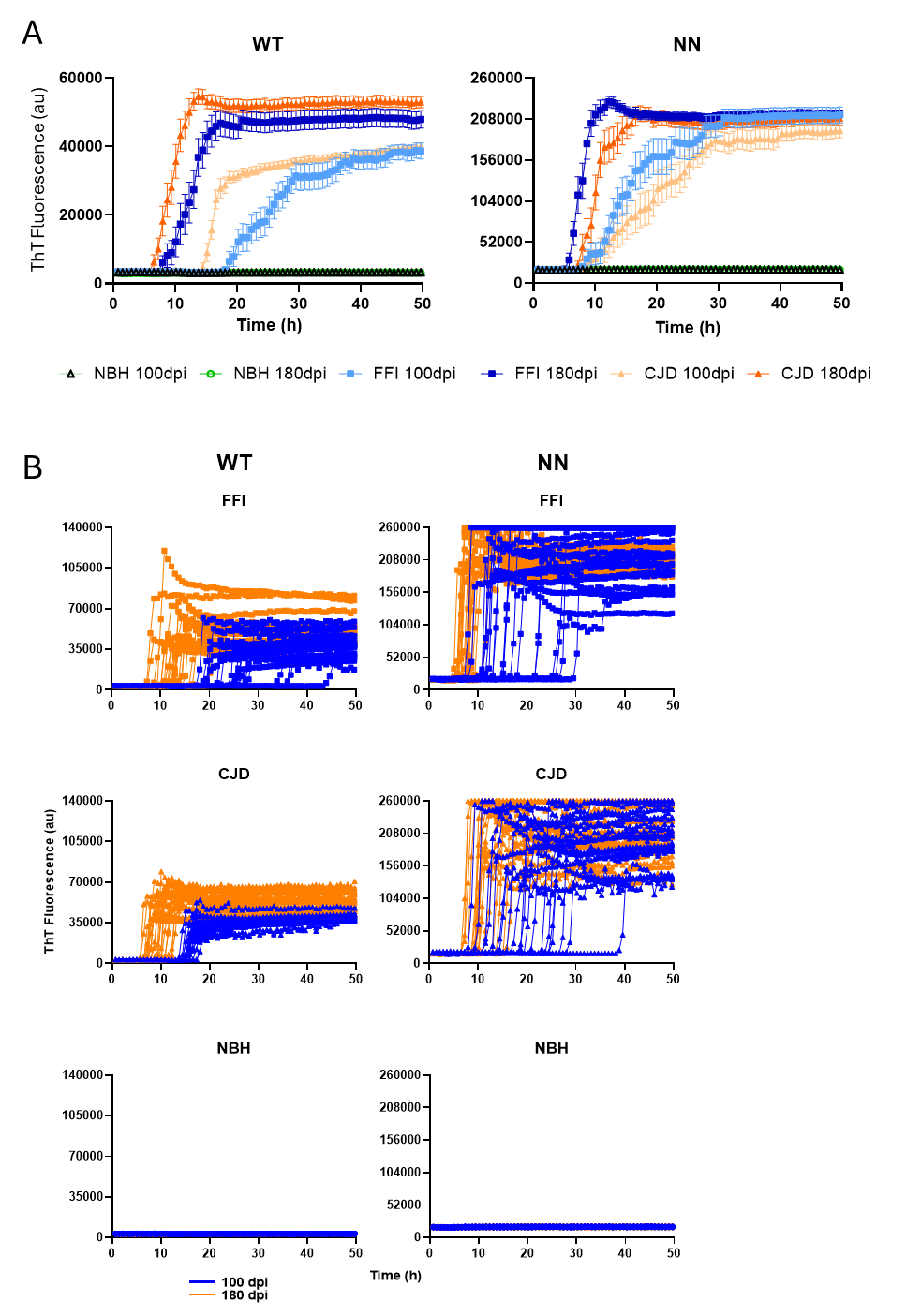

Supplement: Supplementary Info [file NIHMS2113846-supplement-Supplementary_Info.docx]
